# Supplementary material for: Long-Term Evolution of Email Networks: Statistical Regularities, Predictability and Stability of Social Behaviors
Source: PLoS One. 2016 Jan 6;11(1):e0146113. doi: 10.1371/journal.pone.0146113 (PMC4703408; doi:10.1371/journal.pone.0146113)
Supplement: S1 File — (PDF) [file pone.0146113.s001.pdf]

S1 Equivalence between the directed and the undirected network of emails

In our analysis we consider an undirected network of email communication in which we do not differentiate between senders and receivers. Figure A shows that our choice is justified because over the period of one year conversations are bidirectional, that is , except for particular cases, each email receives its answer. The very high Pearson's correlation coefficient of $\rho = 0.83$, $p < 10^{-323}$ confirms the visually apparent linear relationship.

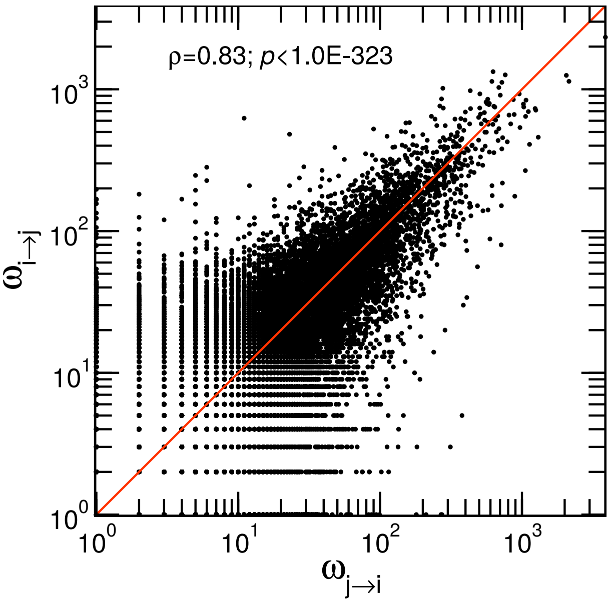

Figure A: **Equivalence of the direct and undirected email network.** Scatter plot of the $\omega_{i\to j}$, the number of emails that user i sends to user j during one year versus $\omega_{j\to i}$. The coordinates of each pair have been randomly given in order to avoid any bias. The red line represents the situation in which all the emails sent were answered.
