# Supplementary material for: Long-Term Evolution of Email Networks: Statistical Regularities, Predictability and Stability of Social Behaviors
Source: PLoS One. 2016 Jan 6;11(1):e0146113. doi: 10.1371/journal.pone.0146113 (PMC4703408; doi:10.1371/journal.pone.0146113)
Supplement: S2 File — (PDF) [file pone.0146113.s002.pdf]

S2 Modeling the distribution of logarithmic growth rates

S2.1 Model selection for the distribution of logarithmic growth rates

In the main text, we analyze the evolution of email communication flows through the logarithmic growth rates of link weights and node strengths (LGRs). We consider the following models for the LGR distributions (Fig. A):

- a Laplace or symmetric exponential distribution (Eq. 3 in the main text)

$$P_{\mathrm{L}}(r|\sigma_{\exp}) = \frac{\exp\left(-|r-\mu|/\sigma_{\exp}\right)}{2\sigma_{\exp}}; \tag{1}$$

- a Gaussian distribution

$$P_{\mathrm{Gauss}}(r|\sigma_{\mathrm{G}}) = \frac{e^{-(r-\mu)^2/2\sigma_{\mathrm{G}}^2}}{\sigma_{\mathrm{G}}\sqrt{2\pi}}; \tag{2}$$

- an asymmetric Laplace distribution

$$P_{\mathrm{asymm-L}}(r|\sigma_{\mathrm{left}}, \sigma_{\mathrm{right}}) = \begin{cases} \frac{\exp(-|r-\mu|/\sigma_{\mathrm{left}})}{\sigma_{\mathrm{left}}+\sigma_{\mathrm{right}}} & \text{if } r \leq 0 \\ \frac{\exp\left(-|r-\mu|/\sigma_{\mathrm{right}}\right)}{\sigma_{\mathrm{left}}+\sigma_{\mathrm{right}}} & \text{if } r > 0 \end{cases}; \tag{3}$$

- a convolution of a Laplace and a normal distribution (Eq. 5 in the main text)

$$P_{conv}(r|\sigma_{\exp}, \sigma_{\mathrm{G}}) = \int_{-\infty}^{\infty} \frac{e^{-|\rho|/\sigma_{\exp}}}{2\sigma_{\exp}} \frac{e^{-(r-\mu-\rho)^2/2\sigma_{\mathrm{G}}^2}}{\sigma_{\mathrm{G}}\sqrt{2\pi}} d\rho. \tag{4}$$

According to the BIC, for any Δt and starting year t, the best fit for the weight LGRs is the convolution of a Laplace distribution and a Gaussian, while for the strength LGRs the best fit is a Laplace distribution (see Fig. A).

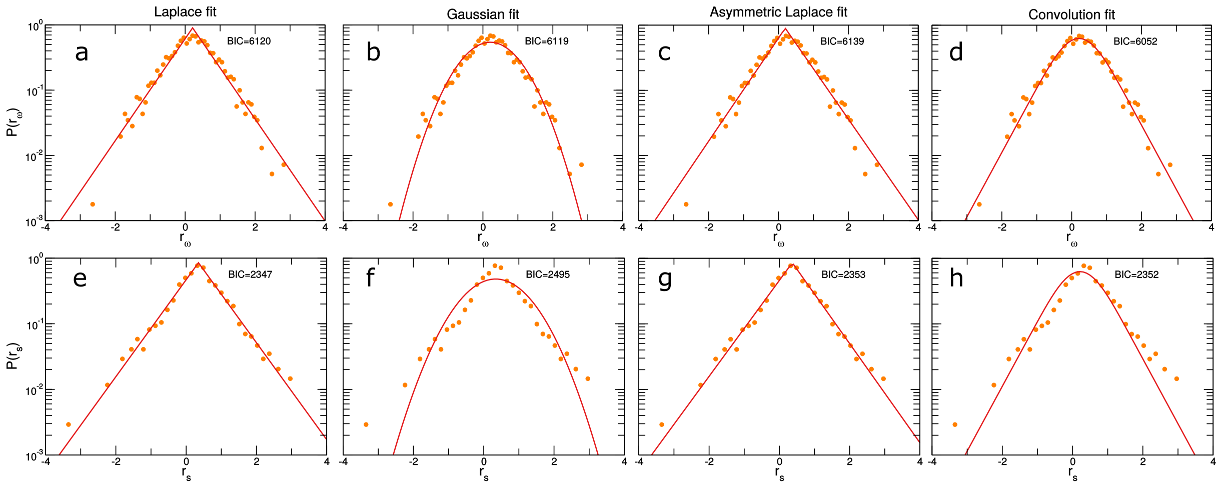

Figure A: **Models for the distributions of weight (top) and strength (bottom) logarithmic growth rates (r_ω and r_s respectively) for $\Delta t = 1$ and $t = 2007$.** Orange circles correspond to $P(r_\omega)$ and $P(r_s)$. Red lines show the the maximum likelihood fits. In the top right of each graph we show the BIC for each fit. (**a**,**e**) Laplace fit according to Eq. (1). (**b**,**f**) Gaussian fit according to Eq. (2). (**c**,**g**) Asymmetrical Laplace fit, according to Eq. (3). (**d**,**h**) Convolution fit according to Eq. (4). We obtain similar results for other starting years t and values of Δt.

S2.2 Stability of the functional form of the logarithmic growth rate distributions

The best fits to the LGR distributions obtained for each starting year $t = 2007, .., 2009$ and $\Delta t = 1, 2, 3$, show that the mode of the distribution changes slightly with starting year (Fig. B), especially for $t = 2007$ and not significant for $t = 2008$ and $\mathrm{t} = 2009$. Therefore in order to assess if the functional form of LGR distributions is stationary (that is with different modes but otherwise with the same model parameters), we need to compare the distribution of centered LGRs, $r^0 = r - \mu(t, \Delta t)$, for fixed Δt. Table A shows the results of comparing pairs of distributions using the Kolmogorov-Smirnov test. According to test results, at a 1% significance level, we cannot reject the hypothesis that for fixed Δt distributions of centered logarithmic growth rates of strengths for different years come from a single distribution. Note that for $\Delta t = 1$ a multiple testing correction to the p-value, would further strengthen our results.

To illustrate the stability of the functional form of $P(r^0_\omega)$ and $P(r^0_s)$ in time, Fig. C shows the weight and strength logarithmic growth rates distributions for each individual year $t = 2007, 2008, 2009$ for $\Delta t = 1$ year, and $t = 2007, 2008$ for $\Delta t = 2$ years (the fit for the aggregate datasets in the same fit as that in Figs. 1B and 2B in the main text).

S2.3 Evolution of model parameters with time

In Figs. 1B and 2B of the main text we show the distribution of the logarithmic growth rates for both weights and strengths. According to the Bayesian Information Criterion (BIC) the best fit is for $P(r^0_\omega)$ a convolution of a Laplace distribution and a Gaussian (Eq. 4) and for $P(r^0_s)$

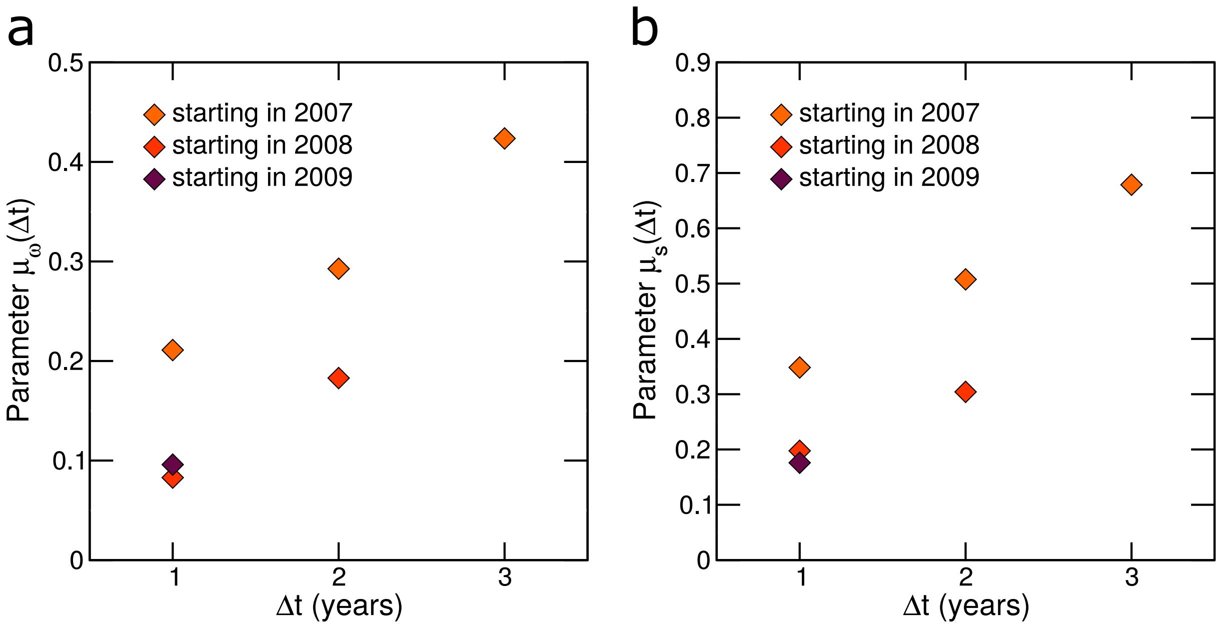

Figure B: **Time evolution of the modes μ for the distribution of logarithmic growth rates.** (**a**,**b**) We show $\mu_\omega(t, \Delta t)$ and $\mu_s(t, \Delta t)$ for $t = 2007, 2008, 2009$ and $\Delta t = 1, 2, 3$.

Comparison Pair $(X,\ \Delta t, (t_1, t_2))$	KS Statistic	p-value
$\omega,\ \Delta t = 1,\ (2007, 2008)$	0.022	0.45
$\omega,\ \Delta t = 1,\ (2008, 2009)$	0.025	0.19
$\omega,\ \Delta t = 1,\ (2007, 2009)$	0.037	0.021
$\omega,\ \Delta t = 2,\ (2007, 2008)$	0.031	0.18
$s,\ \Delta t = 1,\ (2007, 2008)$	0.032	0.63
$s,\ \Delta t = 1,\ (2008, 2009)$	0.031	0.61
$s,\ \Delta t = 1,\ (2007, 2009)$	0.029	0.71
$s,\ \Delta t = 2,\ (2007, 2008)$	0.039	0.42

Table A: Kolmogorov-Smirnov test comparison results. We compare pairs of distributions of centered LGRs for fixed Δt, $(P(r_X^0; t_1, \Delta t), P(r_X^0; t_2, \Delta t))$ for $X = \omega, s$. If the p-value is greater than 0.01, we cannot reject the null hypothesis that both distributions are the same (at a 1% significance level).

a Laplace distribution (Eq. 1). We estimate the parameters using maximum likelihood for the best model in each case (Fig. D).

Figure D shows the changes in the model parameters with Δt. In general we find that as Δt increases, the exponential tails becomes wider and the total density of exchanged emails increases for both weights and strengths. For $P(r_\omega^0)$ the intensity of the Gaussian noise also increases with Δt. The only exception is that for $P(r_\omega^0)$, σ_{exp}^ω seems to stop growing for $\Delta t = 3$.

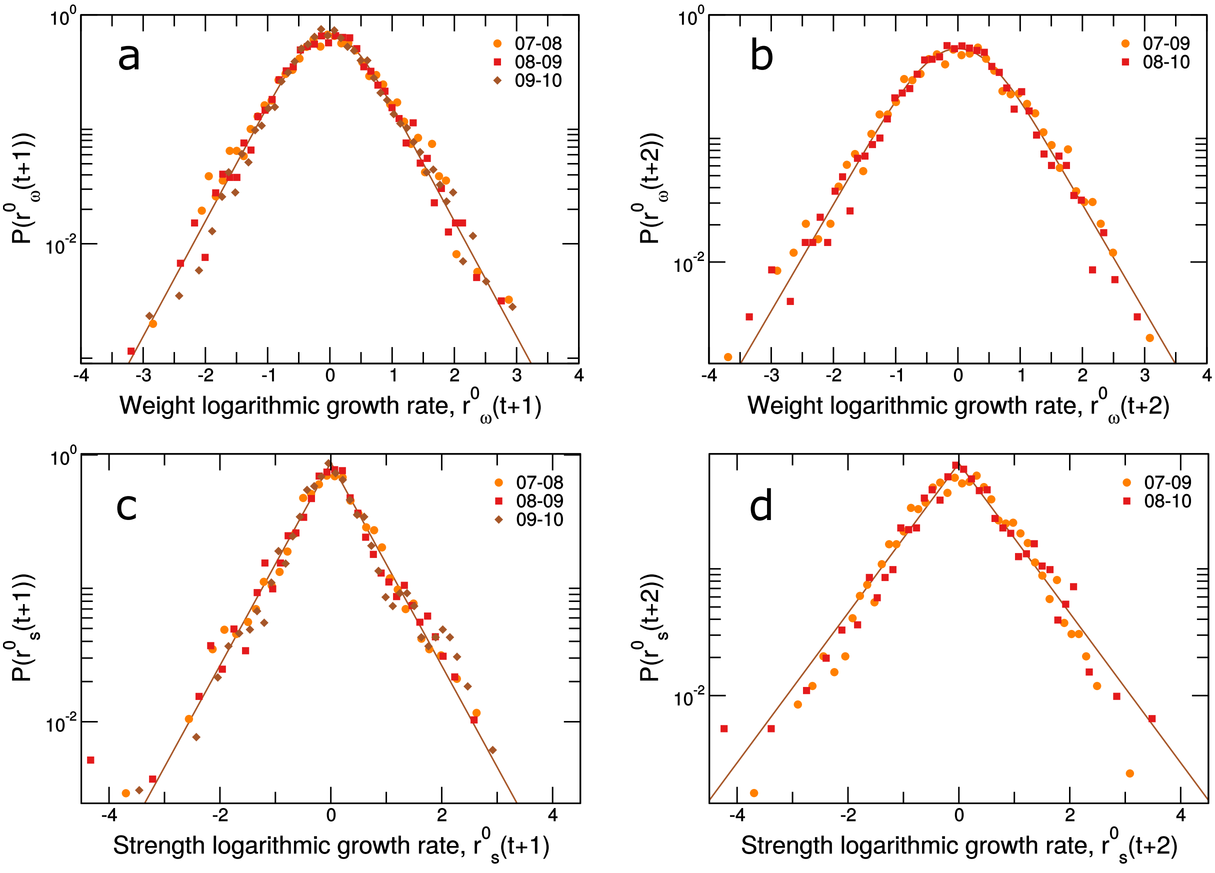

Figure C: **Stability of the distributions of centered logarithmic growth rates.** (**a**,**c**) Centered distributions of the weight (strength) logarithmic growth rates $r^0_\omega(\Delta t = 1)$ ($r^0_s(\Delta t = 1)$) for the years $t = 2007, 2008, 2009$ (dots, squares and diamonds respectively). The line shows the fit to the aggregate of all three years (Figs. 1B and 2B in main text). (**b**,**d**) Distributions of the weight (strength) logarithmic growth rate $r^0_\omega(\Delta t = 2)$ ($r^0_s(\Delta t = 2)$) for the years $t = 2007, 2008$ (dots and squares respectively). The line shows the Laplace fit to the aggregate distribution (Figs. 1B and 2B in main text).

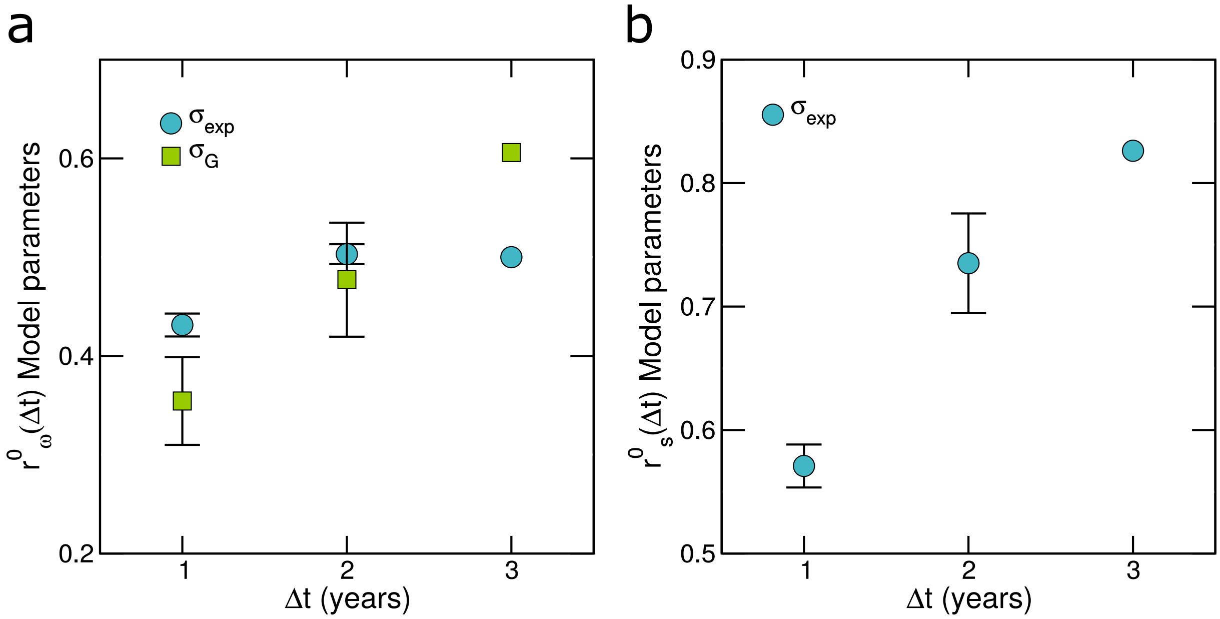

Figure D: **Time evolution of the model parameters for the distribution of logarithmic growth rates.** (**a**) $P(r_\omega^0)$ model parameters estimated from the maximum likelihood of the convolution of a Laplace distribution and a Gaussian, with two parameters σ_{exp}^ω and σ_G^ω (Eq. 4) for $\Delta t = 1, 2, 3$ years. (**b**) $P(r_s^0)$ model parameters estimated from the maximum likelihood of a Laplace distribution, with parameter σ_{exp}^s (Eq. 1) for $\Delta t = 1, 2, 3$ years. The errors are $< 5\%$ in all the cases.
