# Supplementary material for: Long-Term Evolution of Email Networks: Statistical Regularities, Predictability and Stability of Social Behaviors
Source: PLoS One. 2016 Jan 6;11(1):e0146113. doi: 10.1371/journal.pone.0146113 (PMC4703408; doi:10.1371/journal.pone.0146113)
Supplement: S3 File — (PDF) [file pone.0146113.s003.pdf]

S3 Predictability of logarithmic growth rates

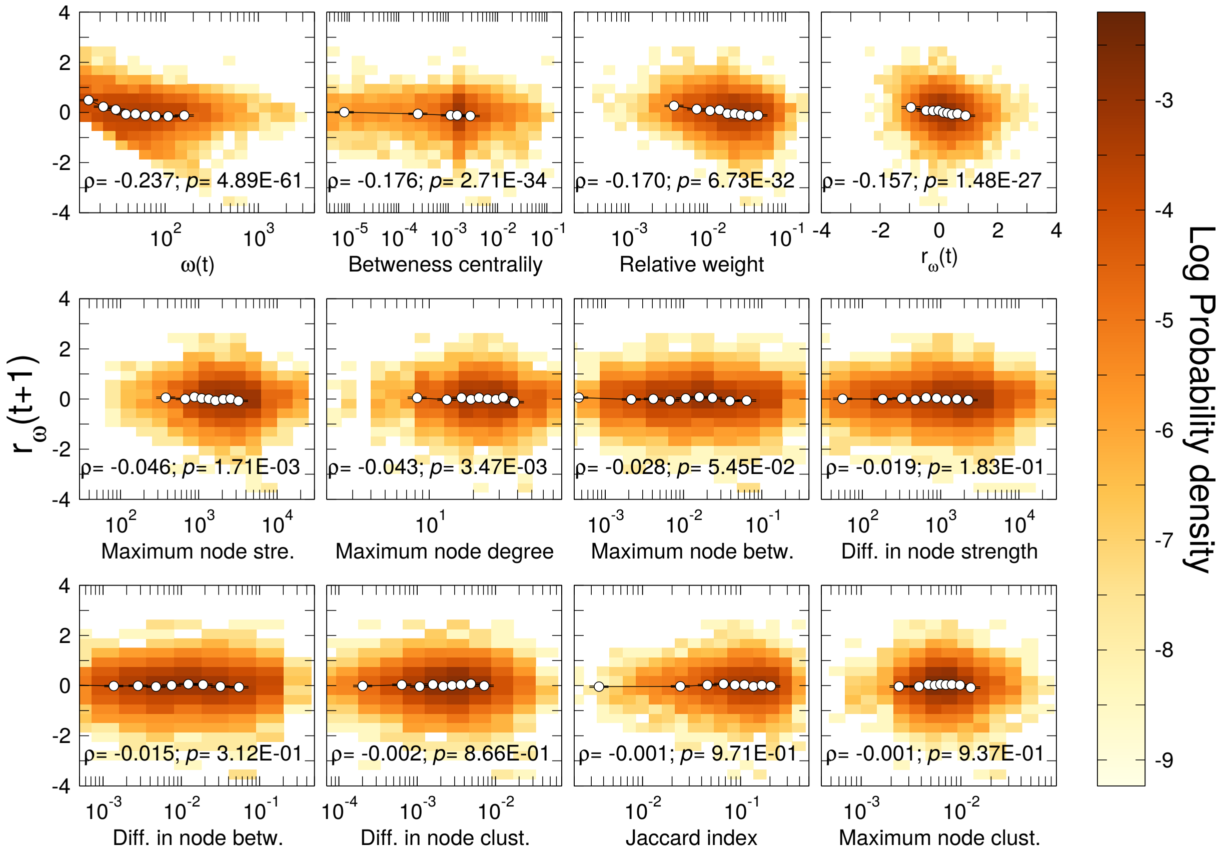

Figure A: **Long-term trends on the weight logarithmic growth rates.** Density plot of the weight logarithmic growth rate $r_\omega(t + \Delta t = 1)$ as a function of the 12 network features mentioned in the text. The lines correspond to the mean and the error of the mean in each bin along the x axis. We show the Spearman's ρ and the significance of the correlation at the bottom of each graph.

Despite the accurate mathematical description of the logarithmic growth rate distributions, in the main text we show that logarithmic growth rates for weight and strengths are highly unpredictable.

$\mathbf{r_\omega(t+1)}$ To assess the predictability of $r_\omega(t+1)$ we analyzed the correlation with a number of network features that we could measure at time t. We choose an array of network features that we thought could bear a relationship with the evolution of communication weights. Specifically, for each edge (i, j) we measured:

- $\omega_{ij}(t)$;
- the betweenness centrality of the edge;

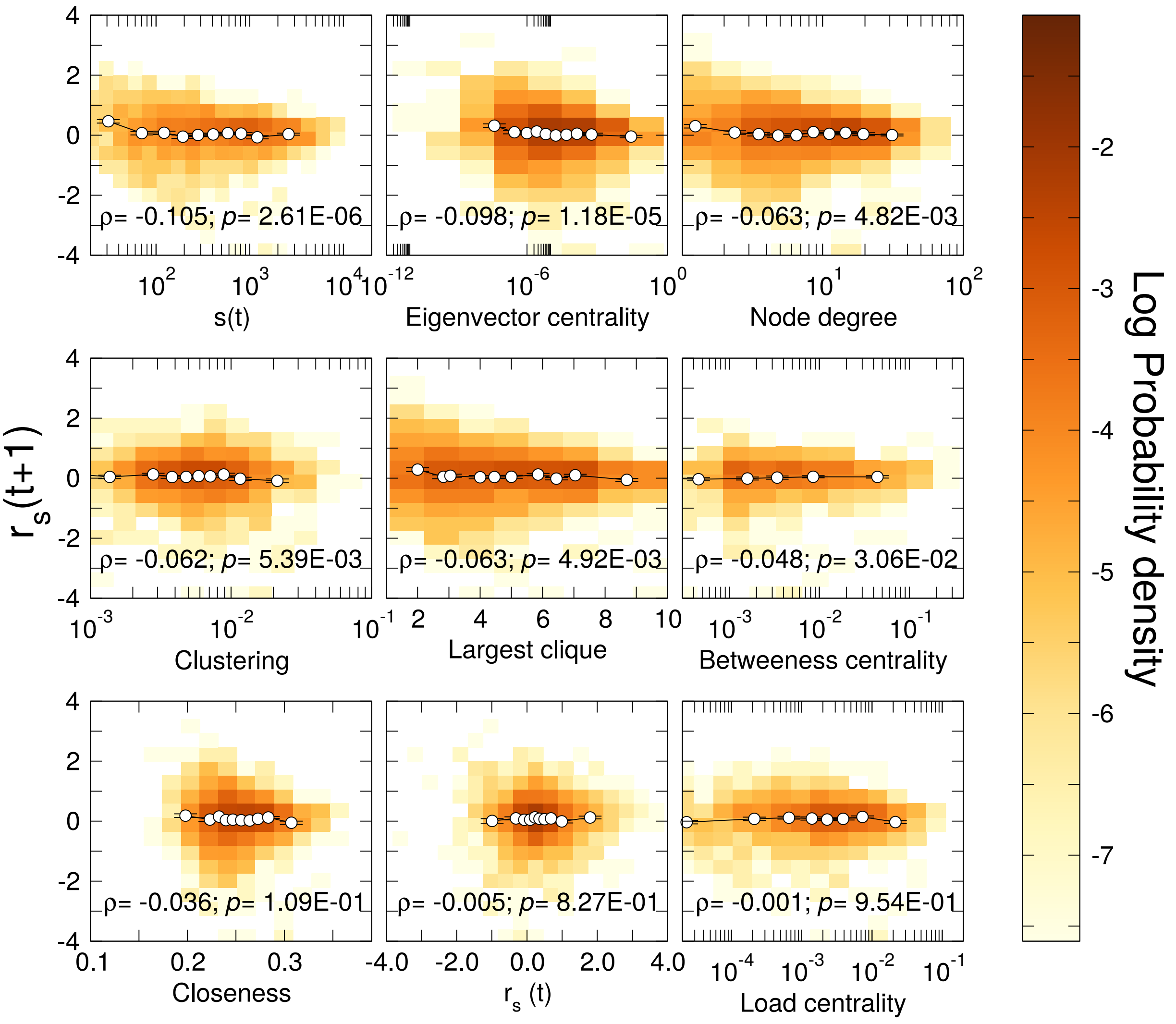

Figure B: **Long-term trends on the strength logarithmic growth rates.** Density plot of the strength logarithmic growth rate $r_s(t + \Delta t = 1)$ as a function of the 9 network features mentioned in the text. The lines correspond to the mean and the error of the mean in each bin along the x axis. We show the Spearman's ρ and the significance of the correlation at the bottom of each graph.

- the relative weight of the edge: $\bar{r}_{ij} = (\omega_{ij} \cdot k_i \cdot k_j)/(s_i \cdot s_j)$;
- $r_\omega^{ij}(t)$;
- the Jaccard index of the edge $J_{ij} = \frac{|\text{neigh}(j) \cap \text{neigh}(i)|}{|\text{neigh}(j) \cup \text{neigh}(i)|}$;
- the maximum node strength $\max\{s_i, s_j\}$;
- the maximum node degree $\max\{k_i, k_j\}$;

- the maximum node betweenness;
- the maximum node clustering;
- the absolute difference in node strength $|s_i - s_j|$;
- the absolute difference in node betweenness;
- the absolute difference in node clustering;

Figure A shows the density plots of $r_s(t+1)$ versus the different features above ordered from higher to lower significance. First of all, we observe that correlations are significant for about half of the features we analyze. However, it is obvious that even for the most significantly correlated feature $\omega(t)$, the variability of $r_\omega(t+1)$ for a fixed value of $\omega(t)$ is too large for this feature to produce accurate predictions (main text Fig. 3E) as indicated by the rather modest value of Spearman's $\rho = -0.24$.

$\mathbf{r_s(t+1)}$ To assess the predictability of $r_\omega(t+1)$ we analyzed the correlation with a number of network features that we could measure at time t. We choose an array of network features that we though could bear a relationship with the evolution of node communication strengths. Specifically, for each node i we measured:

- $s_i(t)$:
- the eigen vector centrality;
- k_i;
- $r_s(t)$;
- the clustering of node i;
- the size of the largest clique containing node i;
- the betweenness centrality of node i;
- the closeness centrality of node i;
- the load centrality of node i, that is the fraction of all shortest paths that connect any pair of nodes in the network that pass through i.

Figure B shows the density plots of $r_s(t+1)$ versus the different features above ordered from higher to lower significance. As for the weights, we observe that correlations are significant for about half of the features we analyze. In the same way, even for the most significantly correlated feature $s(t)$, the variability of $r_s(t+1)$ for a fixed value of $s(t)$ is too large for this feature to produce accurate predictions (main text Fig. 3F). Remarkably, we find that there is not a significant correlation between $r_s(t+1)$ and $r_s(t)$.

Leave-one-out experiments Consider a dataset (x, r) in which x is the network feature and r is the corresponding logarithmic growth rate. In general, we find that we can mathematically model the dependence of r in x. To assess the predictability of logarithmic growth rates from network features at time t, we perform leave-one-out experiments for selected network features. For each point in our dataset (x_i, r_i), we construct a new training dataset in which we remove this point. Then we train our model (that is, we estimate the model parameters) with the training dtatset. Finally, we obtain a prediction p_i of r_i from the trained model using x_i as our input. To estimate the accuracy of the predictions, we compute the mean squared error (MSE), that is MSE= $\frac{1}{N}\sum_{i=1}^{N}(p_i - r_i)^2$. The sample sizes are $N_\omega = 4,721$ and $N_s = 2,013$ for the prediction of $r_\omega(t+1)$ and $r_s(t+1)$, respectively.

The features we consider are (Fig. 3 main text):

- the most significantly correlated features $x = \omega(t), s(t)$, for which we assume that $r = A\ \exp(-r \cdot B) + C$;
- the previous value of the variable we want to predict $x = r_\omega(t+1), r_s(t+1)$ for which we assume $r = A \cdot x + B$;
- the mode of the logarithmic growth rate distributions μ_ω, μ_s which are constants.

Finally, we perform leave-one-out experiments using the Random Forest (main text Fig. 3E-F). For each training dataset, Random Forest uses *all* the features for edges/nodes we have listed previously as inputs to train the algorithm and produce a prediction for the data point not present in the training data set. Our results show that using the Random Forest does not yield significantly better predictions than using using the most correlated features, $\omega(t)$ and $s(t)$for $r_\omega(t+1)$ and $r_s(t+1)$, respectively.
