# Supplementary material for: Long-Term Evolution of Email Networks: Statistical Regularities, Predictability and Stability of Social Behaviors
Source: PLoS One. 2016 Jan 6;11(1):e0146113. doi: 10.1371/journal.pone.0146113 (PMC4703408; doi:10.1371/journal.pone.0146113)
Supplement: S4 File — (PDF) [file pone.0146113.s004.pdf]

S4 Social signature and communication strategies

In Figs. 4 and 5 of the main text we show that the standardized Shannon Entropy S_i and the fraction f_i of all emails exchanged with preexisting contacts define an individual signature and communication strategies that are remarkably stable over several years.

S4.1 Stationarity of distributions

In this section, we demonstrate that distributions for ΔS_i and Δf_i are stationary for a fixed Δt, that is independent of the starting year $t = 2007, 2008, 2009$. We perform a Kolmogorv-Smirnov (KS) test to compare pairs of distributions. The results in Table A show that at a 1% significance level, we cannot reject the null hypothesis that for fixed Δt then distribution for different starting years t come form a unique distribution (Figs. A and B). Therefore, because of this stationarity we can pool data for different years together to perform the time evolution analysis we show in Figs. 4 and 5 of the main text.

Comparison Pair $(X, \Delta t, (t_1, t_2))$	KS Statistic	p-value
ΔS_i, $\Delta t = 1$, $(2007, 2008)$	0.044	0.35
ΔS_i, $\Delta t = 1$, $(2008, 2009)$	0.044	0.28
ΔS_i, $\Delta t = 1$, $(2007, 2009)$	0.028	0.86
ΔS_i, $\Delta t = 2$, $(2007, 2008)$	0.071	0.03
Δf_i, $\Delta t = 1$, $(2007, 2008)$	0.071	0.023

Table A: Kolmogorov-Smirnov test comparison results. We compare pairs of distributions of $\Delta S_i(\Delta t, t)$ and $\Delta f_i(\Delta t, t)$ for fixed Δt. If the p-value is greater than 0.01, we cannot reject the null hypothesis that both distributions are the same (at a 1% significance level).

S4.2 Social signatures and communication strategies of individuals are stable over time

In here, we reproduce the analysis in Figs. 4C and 5C of the main text for the self and reference absolute differences of both S_i and f_i for different starting years $t = 2007, 2008, 2009$. We find that results for separate years are consistent with results for aggregate data: social signatures and communication strategies of individuals are stable over time.

S4.3 Analysis of the size effect in social signature measurements

The social signature of an individual measures the way in which a user distributes the communication flow among its contacts. In our analysis, we thought about two different measures to quantify how the social signature of a user changes in time: the Gini coefficient and the standardized Shannon entropy.

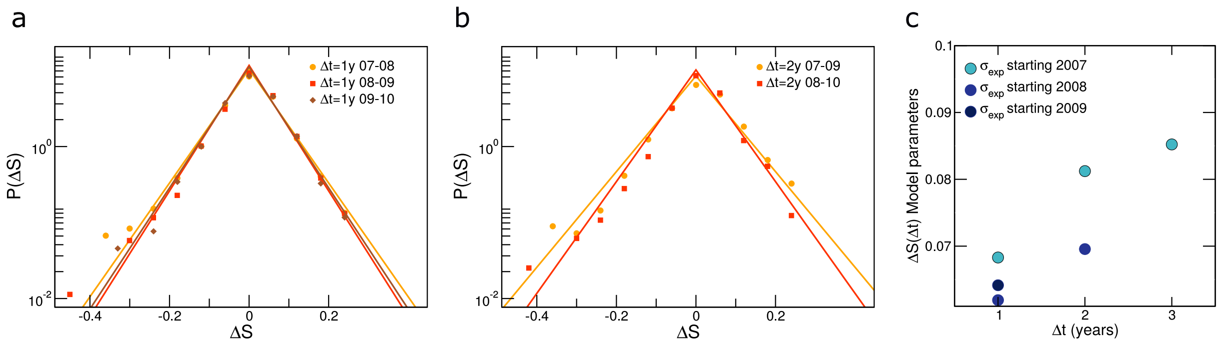

Figure A: **Stationarity of the changes of the standardized Shannon entropy of individuals.** (**a**) Distributions of the change of individual standardized Shannon entropy $\Delta S_i(\Delta t = 1, t) = S_i(t+1) - S_i(t)$, $\forall i$ for $t = 2007, 2008, 2009$ (dots, squares and diamonds, respectively). Lines show Laplace distribution fits ($P(\Delta S_i) = \exp(-|\Delta S_i|/\sigma)$, $\mu = 0$; for $t = 2007 : \sigma = 0.068$, for $t = 2008 : \sigma = 0.062$, and for $t = 2009 : \sigma = 0.061$). (**b**) Distributions of the change of individual standardized Shannon entropy $\Delta S_i(\Delta t = 2, t)$, $\forall i$ for $t = 2007, 2008$ (dots and squares, respectively). Lines show Laplace distribution fits (for $t = 2007 : \sigma = 0.081$ and for $t = 2008 : \sigma = 0.070$). (**c**) Laplace parameters estimated via likelihood maximization: σ ($\mu = 0$) for the distributions in (a) and (b), and σ for $P(\Delta S(\Delta t = 3))$.

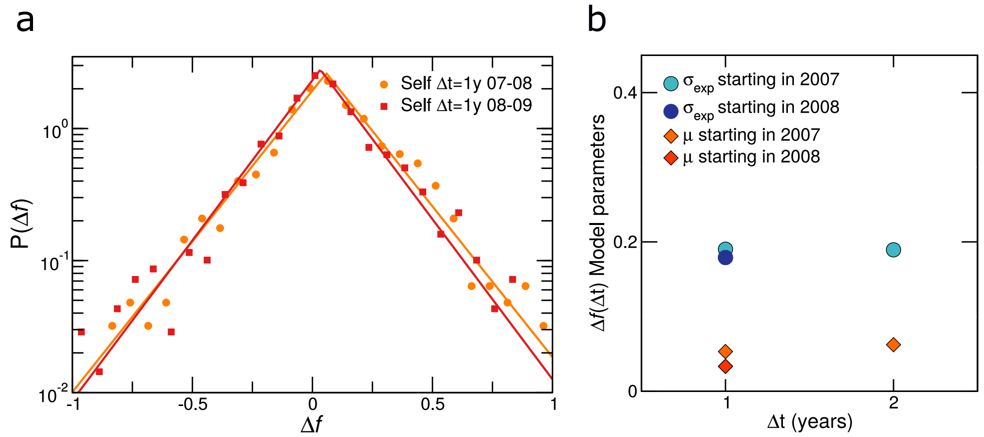

Figure B: **Stationarity of the changes in the fraction of emails.**(**a**) Distributions of the change in the fraction of emails sent by users to pre-existing contacts $\Delta f_i(\Delta t = 1, t)$ for $t = 2007, 2008$ (dots and squares, respectively). Lines show fits to Laplace distributions ($P(\Delta f_i) = \exp(-|\Delta f_i - \mu|/\sigma)$; for $t = 2007 : \sigma = 0.19$, $\mu = 0.053$ and for $t = 2008$: $\sigma = 0.17$, $\mu = 0.033$). (**b**) Laplace parameters estimated via likelihood maximization: σ and μ for the distribution in (a) and σ from $P(\Delta f(\Delta t = 2))$.

The Gini coefficient 1 which is used to measure inequalities in wealth distributions within and across countries, measures the disparity of weights between the different connections of

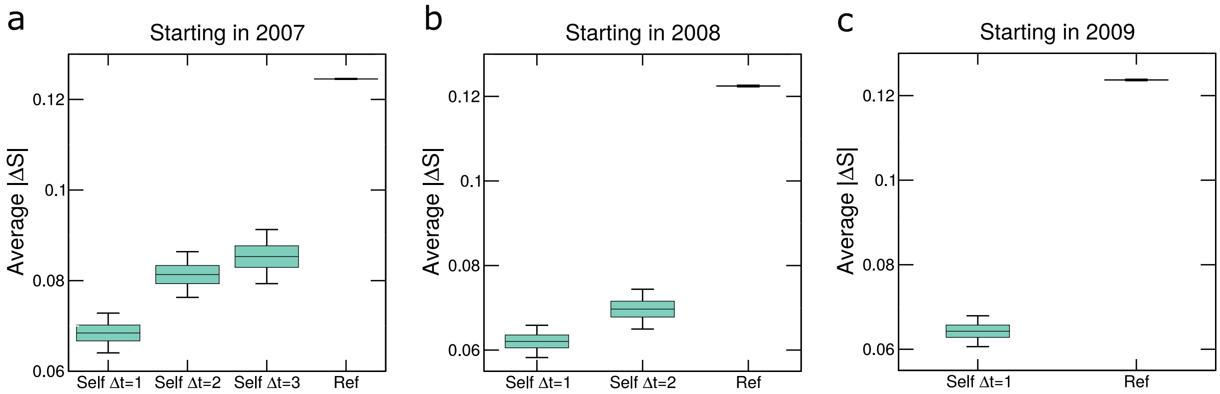

Figure C: **Time evolution of social signatures.** (**a, b, c**) Comparison between the absolute difference in individual social signatures $|\Delta S_i(\Delta t, t)|_{\text{self}} = |S_i(t + \Delta t) - S_i(t)|$ and the typical absolute difference of entropies between individuals $|\Delta S_{ij}(t)|_{\text{ref}} = |S_i(t) - S_j(t)|$ for $t = 2007$ in (a), $t = 2008$ in (b) and $t = 2009$ in (c). The width of the boxes and the bars correspond to one and two standard errors of the mean, respectively.

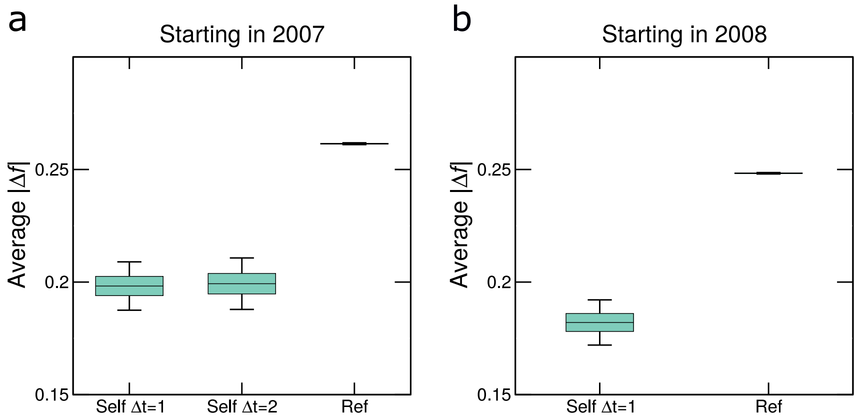

Figure D: **Time evolution of communication strategies.** (**a, b**) Comparison between the absolute difference in individual social signatures $|\Delta f_i(\Delta t)|_{\text{self}} = |f_i(t + \Delta t) - f_i(t)|$ and the typical absolute difference of entropies between individuals $|\Delta f_{ij}(t)|_{\text{ref}} = |f_i(t) - f_j(t)|$ for $t = 2007$ in (a) and $t = 2008$ in (b). The width of the boxes and the bars correspond to one and two standard errors of the mean, respectively.

each individual. In our case, we compute the Gini coefficient as :

$$G_i = \left| 1 - \frac{2}{k_i} \sum_{z=2}^{k_i} X_z^i \right|, \tag{1}$$

where k_i is the number of contacts of user i, and $\mathbf{X}^i$ is the cumulative proportion of emails exchanged by user i and her contacts when we order contacts by increasing fraction of exchanged emails. Therefore, $X_z^i = \sum_{c=1}^{z} f_c$ with f_c the fraction of emails exchanged with contact in position c. If the Gini coefficient is equal to one, then the distribution i very unequal, with most

of its weight in a single channel. If the Gini coefficient is equal o zero, then, all the channels have the same flow.

The Shannon entropy is another measure that quantifies whether the distribution of the flow communication among contacts is even or not. In our case, we use the standardized Shannon entropy so that it takes positive values between zero and one:

$$S_i = \frac{-\sum_{j=1}^{k_i} \frac{\omega_{ij}}{s_i} \log \frac{\omega_{ij}}{s_i}}{\log k_i}, \tag{2}$$

where k_i is the number of contacts of user i. Note that $S_i = 1$ when user i exchanges the same number of emails with all her contacts and $S_i \approx 0$ when she exchanges almost all of her emails with a single contact.

While these two measures are normalized, it is not clear whether they show a systematic trend as we increase the number of contacts. To assess the effect of the number of contacts, for a given number of contacts with values from $c = 3$ to $c = 20$, we measure the average Gini coefficient and the standardized Shannon entropy over $N = 10000$ randomizations of the weights (see Fig.E). While the average of the standardized Shannon entropy increases slightly and progressively, taking a gap in the average of 0.015 up to 20 contacts, the Gini coefficient has larger increase for a small number of contacts until its arrive to a plateau at 20 contacts, with a gap in the average of the Gini coefficient of 0.27 between $c = 3$ and $c = 20$.

Note, that since the majority of users have few contacts (90% of users have less than 20 contacts), the variation of the standardized Shannon entropy in that range is very small.

S4.4 Communication strategies: analysis of the contact turnover of users

In the main text, we analyze the evolution of the communication strategy by measuring the fraction of emails sent in year t to contacts that existed in year $t - 1$. Alternatively, we can measure the turnover in the network contacts and analyze whether we find a similar behavior.

Specifically, we measure the fraction f_{k_i} of contacts with whom user i exchanged contacts in years t and $t - 1$ compared to the total number of contacts k_i during year $t - 1$

$$f_{k_i}(t) = \frac{k_i(t) \cap k_i(t-1)}{k_i(t)}. \tag{3}$$

Therefore, if $f_{k_i}(t) = 1$, user i has maintained all her contacts from the previous year (regardless of the number of emails mail that has exchanged with each one of them), whereas $f_{k_i}(t) = 0$, user i has changed all her contacts.

The distribution of f_k (Fig. Fa) indicates that most individuals tend to maintain the majority of their contacts from year to year. The several peaks of the distribution are due to the discrete number of values that fractions can take on those users with few contacts (52% of users have less than 5 contacts). Although the distribution is irregular, the mass of the distributions is slightly concentrated for $f_k > 0.5$, with a 62% of the mass at $f_k > 0.5$.

To study the stability of each individual's turnover in the long term, we measure the change $\Delta f_{k_i}(\Delta t) = f_{k_i}(t + \Delta t) - f_{k_i}(t)$ at $\Delta t = 1$ year and $\Delta t = 2$ years (Fig. Fb). From the

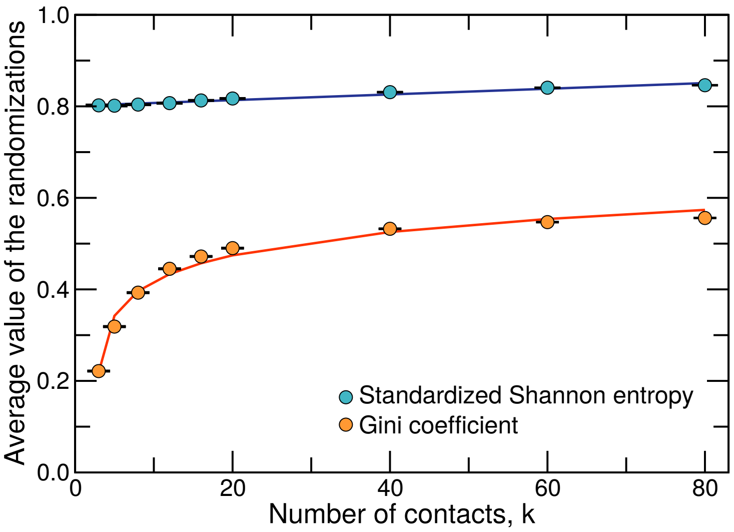

Figure E: **Size effect of the standardized Shannon entropy and the Gini coefficient.** For a fixed number of contacts, we generate $N = 10000$ random samples using link weights from our data. We show the average of the standardized Shannon entropy and the Gini coefficient (blue and orange dots respectively) as a function of the number of contacts. The lines show the best fit of the average: a linear fit for the standardized Shannon entropy (in dark blue, $f(\bar{S}) = 0.80 + 0.0062 \cdot \bar{S}$) and a logarithmic fit for the Gini coefficient (in red, $f(\bar{G}) = 0.28 + 0.66 \cdot ln(\bar{G} - 2.63)$).

distribution of these changes we observe that most users do not change their communication strategy from year to year (the mode is at $\Delta f_k(\Delta t) = 0$). However, 11% of the individuals change their communication strategy by $|\Delta f_k(\Delta t)| > 0.5$.

Despite this variability, we find that, on average, an individual's turnover is stable in the long term (Fig. Fc). In particular, we compare the absolute individual change $|\Delta f_{k_i}(\Delta t)|_{\text{self}} = |f_{k_i}(t + \Delta t) - f_{k_i}(t)|$ with the typical absolute difference between individuals $|\Delta f_{k_{ij}}|_{\text{ref}} = |f_{k_i}(t) - f_{k_j}(t)|$, $\forall j \neq i$. We observe that the yearly variation of an individual's communication strategy is typically much smaller (even when $\Delta t = 2\,\text{years}$) than the variation between individuals, confirming the existence of persistent turnover strategies even at the scale of several years.

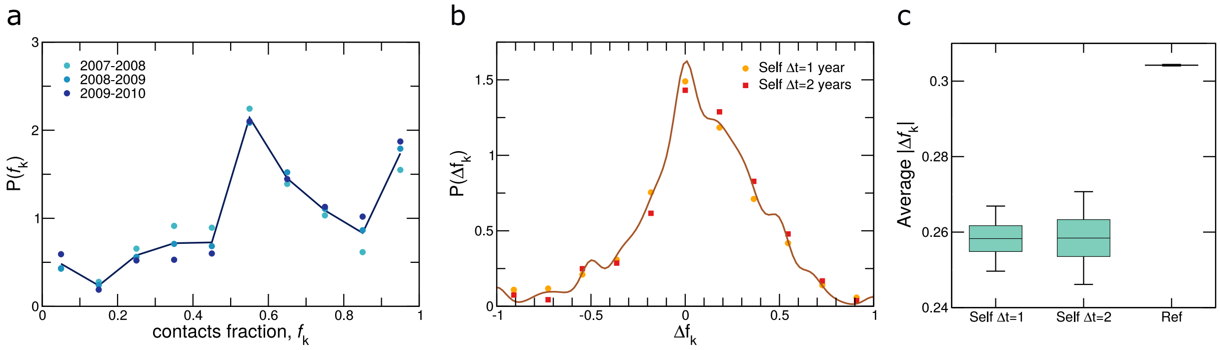

Figure F: **Variability of individual turnover of contacts.** (**a**) Distributions of the fraction of contacts that are preserved in two consecutive years $f_{k_i}(t)$ (see Eq.3). The line shows the distribution for the aggregate of the three datasets. Note that the distributions are stable in time. (**b**) Distributions of $\Delta f_{k_i}(\Delta t) = f_{k_i}(t + \Delta t) - f_{k_i}(t)$, the change in the fraction of preserved users, for $\Delta t = 1, 2$ years (dots and squares respectively). Note that the distributions are stable in time. The line shows the smooth kernel distribution for the the two distributions pooled together. (**c**) Boxplot of the average over all the users of the absolute differences in the fraction of preserved contacts of each user (self) $|\Delta f_{k_i}(\Delta t)|_{self} = |f_{k_i}(t + \Delta t) - f_{k_i}(t)|$ for $\Delta t = 1, 2$ years compared to the difference in the fraction of preserved contacts between a random pair of users (reference) $|\Delta f_{k_{ij}}|_{ref} = |f_{k_i}(t) - f_{k_j}(t)|$. Note that users have stable communication strategy since the difference in the fraction of preserved contacts for an individual is small compared to the average difference between pairs of users.
